# Synergy in Aqueous Systems Containing Bioactive Ingredients of Natural Origin: Saponin/Pectin Mixtures

**DOI:** 10.3390/polym14204362

**Published:** 2022-10-16

**Authors:** Hristina Petkova, Ewelina Jarek, Mitko Doychinov, Marcel Krzan, Elena Mileva

**Affiliations:** 1 Institute of Physical Chemistry, Bulgarian Academy of Sciences, “Acad. G. Bonchev” Str. Bl. 11, 1113 Sofia, Bulgaria; 2 Jerzy Haber Institute of Catalysis and Surface Chemistry, Polish Academy of Sciences, 8 “Niezapominajek” Str., 30239 Krakow, Poland

**Keywords:** *Quillaja* saponins, *Apple* pectins, surface tension, surface dilatational rheology, gel formation, thin liquid films

## Abstract

Biocompatible and biodegradable ingredients of natural origin are widely used in the design of foam and emulsion systems with various technological applications in the food, cosmetics and pharmaceutical industries. The determination of the precise composition of aqueous solution formulations is a key issue for the achievement of environmentally-friendly disperse systems with controllable properties and reasonable stability. The present work is focused on the investigation of synergistic interactions in aqueous systems containing *Quillaja* saponins and *Apple* pectins. Profile analysis tensiometer (PAT-1) is applied to study the surface tension and surface dilational rheology of the adsorption layers at the air/solution interface. The properties and the foam films (drainage kinetics, film thickness, disjoining pressure isotherm, critical pressure of rupture) are investigated using the thin-liquid-film (TLF) microinterferometric method of Scheludko–Exerowa and the TLF-pressure-balance technique (TLF-PBT). The results demonstrate that the structure and stability performance of the complex aqueous solutions can be finely tuned by changing the ratio of the bioactive ingredients. The attained experimental data evidence that the most pronounced synergy effect is registered at a specific saponin:pectin ratio. The obtained information is essential for the further development of aqueous solution formulations intended to achieve stable foams based on mixtures of *Quillaja* saponins and *Apple* pectins in view of future industrial, pharmaceutical and biomedical applications.

## 1. Introduction

The present work is focused on the investigation of complex aqueous systems containing the biocompatible and biodegradable ingredients of natural origin, namely *Quillaja* saponins and *Apple* pectins. While there is a large number of investigations that deal with studies on the properties of solutions based on a single component, either saponin-only (e.g., [1,2,3,4,5,6,7]) or pectin-only (e.g., [8,9]), to the best of our knowledge, there are none dealing with compositions based on saponin/pectin mixtures.

Saponins are natural compounds extracted from plant resources [1,2,3,4]. They belong to a large group of glycosides and are usually classified based on their structural diversity. Saponins consist of a hydrophobic aglycone structure (triterpenoid or steroid) and one to three hydrophilic sugar fragments [2]. Because of the hydration of the sugar portions, saponin molecules are soluble in aqueous systems. The specific amphiphilic configuration of the molecules enables them to adsorb at the water/air interface and determines their action as surface active compounds. It was established that depending on various plant origins and structures, the critical micellar concentration in ambient conditions may vary in the interval of 0.013 ± 0.7 g/L [7]. The origin and structural diversity of the saponins are crucial for their biological activities. These substances may act as antiallergic, antifungal, anti-inflammatory, antimicrobial, cytotoxic and hypocholesterolemic compounds, while some species reduce fat and cholesterol adsorption, inhibit active nutrient transport, etc. [1,5].

Pectins belong to a family of plant-originated oligo- and polysaccharides and are considered the most complex polysaccharides encountered in nature [8,9,10,11,12,13]. Although various compounds from the pectin group have common structural peculiarities, their fine molecular organization is very diverse. Pectins are heterogeneous polysaccharides composed mainly of homogalacturonan (HG—linear chain of 1,4-linked α-D-galacturonic acid (α-D-GalA) residues which are partially methyl-esterified at C-6 carboxyl groups), rhamnogalacturonan I (RG-I—highly branched and having a backbone of alternating α-L-rhamnose (Rha) and α-D-GalA residues), and rhamnogalacturonan II (RG-II—very complex portion and its detailed chemical structure has not been established) [9,10]. The pectin molecules usually contain about 65% HG, 20–35% RG-I and up to 10% RG-II [10].

Despite the different origins and structures, pectins exhibit similar physicochemical properties and human health benefits, such as lowering cholesterol and glucose levels and stimulating the immune response [10,11]. These substances are also reported to act as strong gelating agents [14,15,16,17,18,19]. The 3D-bulk network of the polymer chains is held together by weak interactions, e.g., hydrogen bonds, electrostatic and hydrophobic interactions. The mechanisms of gelation, as well as the network stability, depend on the pH of the system, temperature and degrees of esterification (DE) of the carboxylic groups of the galacturonic acid constituents. For example, high methoxy pectin gels (HMP, DE > 50%) are usually held together by intermolecular hydrogen bounds and by hydrophobic bonding between methyl-esters, while low methoxy-pectin gels (LMP, DE < 50%) are stabilized in the presence of, e.g., Ca^2+^ via ionic cross-linkage between the carboxylic groups of the different chains [10,14,15]. The interactions with some other constituents of natural origin have also been investigated [19,20].

Due to their natural origin and biological functionalities, pectins and saponins are commonly used in the pharmaceutical and food industries. Thus, the surface activity of saponins and their foaming properties define their wide applications as foam stabilizers [2]. Despite their intrinsic amphiphilic structure, pectins are not reported to dramatically reduce the surface or the interfacial tension [21] except in cases of ultrahigh methoxylated compounds [22]. However, because of their gel-creating capacity in aqueous media, they are often used as oil-in-water emulsion stabilizers.

The present paper aims to report the results from investigations of the properties of aqueous solution formulations based on saponin and pectin mixtures. The synergistic interactions of the components and the fine-tuning of the systems’ properties are studied in view of favoring the future formation of biodegradable and biocompatible foams with potential applications in the pharmaceutical and food industries. The research is focused on the details of the adsorption layer properties at the air/solution interface, as well as the microscopic foam films. These are viewed as two main pillars linked to the stability of foam systems based on the saponin/pectin mixtures. The goal is to establish the impact role of well-detectable synergistic interactions appearing in these complex fluid formulations and to outline design approaches of the mixed systems aimed at possible applications meant for stabilizers of industrial foams.

## 2. Materials and Methods

The *Quillaja* saponins used in the present study were purchased from VWR International, Gdańsk, Poland (Saponin, Reagent Grade catalog number VWR0163, LOT 1666C426) in the form of a white powder and represent an extract from *Quillaja* saponaria bark, in which the saponins content is larger than 80%. These *Quillaja* saponins are bidesmosidic triterpenoid compounds [2,3]. The investigated extract is a mixture of different components with molecular mass between 1070 and 1700 g/mol (1.0–1.7 × 10^3^ g/mol) and an average molecular mass of 1650 g/mol; the estimated critical micellar concentration (CMC) in water at ambient conditions is ~0.65 g/L [23].

The *Apple* pectins applied in the present investigation were purchased from Sigma-Aldrich, Darmstadt, Germany (Pectin from apples, Product number 93854, CAS Number 9000-69-5). The product is in the form of white powder and is characterized by a high degree of esterification (HMP, DE = 50–75%). The molecular mass for *Apple* pectins is in the range of ~1 × 10^5^ g/mol [12,18].

Insofar as both substances are commercial samples with non-strictly defined molecular mass, their concentration is expressed as grams per liter (g/L). Stock solutions were obtained by dissolving each of the compounds in doubly distilled (2D) water with electro-conductivity χ = 1 μS/cm. The initial concentrations were: C(Sp) = 1 g/L for saponin-only, with pH = 5.4 and χ = 170 μS/cm; C(Pc) = 2 g/L for pectin-only, with pH = 3.67 and χ = 120 μS/cm. The mixture of aqueous solutions was prepared using initial single-component systems, which were then mixed in various ratios. Single-component and mixed samples are prepared by diluting the initially prepared stock solutions. The range of investigated *Quillaja* saponins concentrations was 0.025−0.1 g/L and of *Apple* pectins was 0.025−1.0 g/L. All experiments were carried out with freshly obtained single-component or mixture solutions and the temperature during the measurements was kept at 22 °C.

The initial surface tension values were obtained using Tensiometer K20 (KRÜSS, Hamburg, Germany), based on the Wilhelmy-plate method. The dynamic surface tension, as well as the surface dilational rheology of the adsorption layers, are studied by Profile Analysis Tensiometer (PAT-1, Sinterface, Berlin, Germany) [24,25,26]. The experiments were performed in a rising-bubble mode. Surface tension values were achieved via a fitting procedure based on the Young–Laplace equation. Following a thermal equilibration for 20 min using Thermostat LAUDA (Lauda-Königshofen, Germany), surface tension values were registered in the course of 24 h to ensure that equilibrium states were achieved. Then, sinusoidal oscillations of the bubble’s surface were executed at frequencies within the interval of 0.005–0.2 Hz.

The microinterferometric method of Scheludko–Exerowa [27,28,29,30,31,32] and the thin liquid-film–pressure balanced technique (TLF-PBT) [29,30,31,32,33,34,35] was applied for the investigation of the properties and stability of microscopic foam films. Film drainage evolution was studied using the Scheludko–Exerowa cell. The radius of the film holder was r = 2 mm, and the cell allowed film investigations under constant capillary pressure. The TLF-PBT experiments were performed in a porous-plate cell with a pore diameter of ~40 µm [34,35]. Through this technique, disjoining pressure isotherms were obtained (disjoining pressure (Π) against foam-film thickness values (h)) through the controlled increase of the pressure in the measuring cell. Π vs. h studies supply information about the surface forces acting in the films and allow the screening of the film drainage evolution within a wide range of pressures. The critical pressure of the film rupture (P_cr.film_) was achieved by the smooth increase of the pressure in the measuring cell; it is a reliable parameter for the characterization of the microscopic foam film stability [34,35]. All film experiments were conducted at a temperature of 22 °C. Before each measurement, the investigated samples were kept in the measuring cell for half an hour to be thermally equilibrated at the required temperature.

Measurements of the pH and of the electro-conductivity for the investigated solutions were performed by means of inoLab pH 730 pH meter (accuracy ± 0.01) and inoLab Cond 730 conductometer (accuracy ± 0.5%), Wissenschaftlich–Technische Werkstätten GmbH, Weilheim, Germany.

## 3. Results and Discussion

### 3.1. Adsorption Layer Properties

#### 3.1.1. Surface Tension

Due to their amphiphilic properties, saponins tend to adsorb at the air/aqueous solution interface and exhibit significant surface activity [2]. To verify this notion for the particular *Quillaja* saponins investigated here, some initial surface tension measurements were conducted applying Tensiometer K20, KRÜSS.

**Figure 1:**
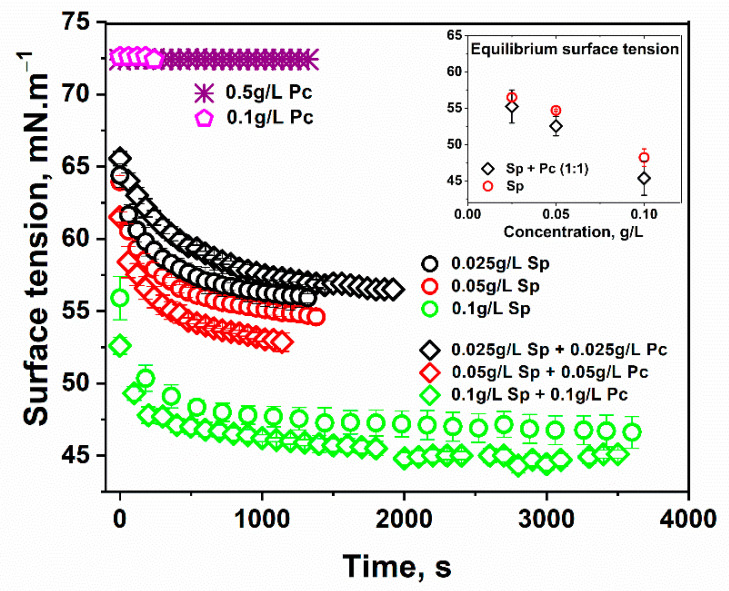
Surface tension of aqueous solutions containing *Quillaja* saponin-only, *Apple* pectin-only, and *Quillaja* saponin and *Apple* pectin 1:1 (g/L, by weight) mixtures, using Tensiometer K20, Krüss. The temperature was kept at 22 °C. Inset: Equilibrium surface tension values of *Quillaja* saponins (red symbols) and *Quillaja* saponin and *Apple* pectin mixture solutions (black symbols).

The surface tension values at the air/solution interface for saponin-only, pectin-only aqueous solutions and mixtures of both compounds at a 1:1 concentration range (g/L, by weight) are presented in Figure 1. A significant initial decrease of the surface tension values is observed within the time range of 0.5–1.5 h upon the increase of the saponin concentration in *Quillaja* saponin-only samples. The time run of the surface tension for *Apple* pectin-only samples at concentrations C(Pc) = 0.1 g/L and C(Pc) = 0.5 g/L shows that this compound does not demonstrate any surface activity.

In the case of a 1:1 mixture (g/L, by weight), however, the impact of *Apple* pectins in short-time measurements is particularly evident at higher concentrations, namely at C(Pc) = 0.05 g/L and C(Pc) = 0.1 g/L. In general, upon the addition of *Apple* pectins, the equilibrium surface tension values of the mixed aqueous samples became lower than for saponin-only solutions. In view of the considerable difference in the mean molecular weight of the components (~1:100), one might advance the hypothesis that saponin molecules are preferentially adsorbed at the interface in short times, and they should be the major reason for the reduction of the surface tension in mixed systems. On the other hand, as presented in the inset of Figure 1, the effects due to possible synergistic saponin–pectin interactions are also evidenced, namely an additional lowering of the obtained ‘equilibrium’ values. Thus, at the increase of the C(Sp), this combined effect leads to a more distinguishable difference as compared to the saponin-only solutions.

In order to clarify the origin of possible synergetic interactions expected to occur in the investigated systems, detailed investigations of the dynamic surface tension and the surface dilational rheology of the adsorption layers at the air/solution interface were performed using the Profile Analysis Tensiometer PAT-1.

**Figure 2:**
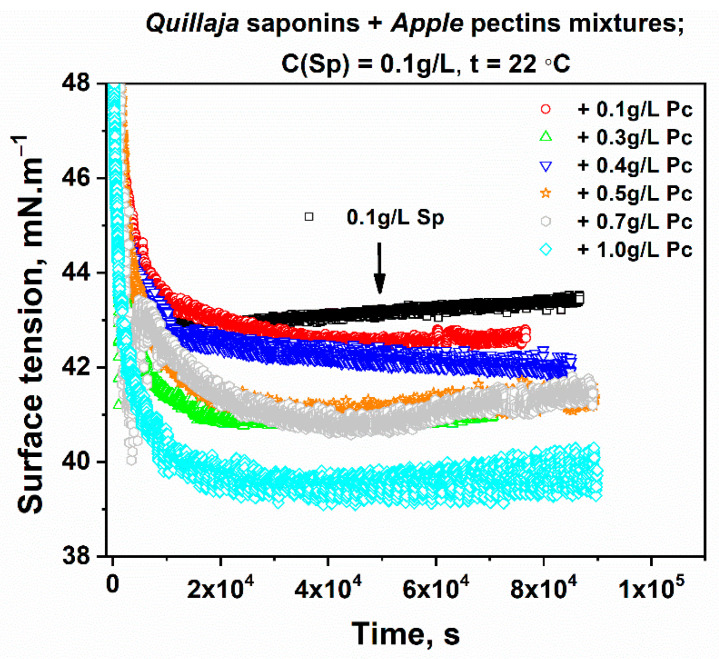
Dynamic surface tension of *Quillaja* saponin and *Apple* pectin mixture solutions at a constant saponin concentration (C(Sp) = 0.1 g/L) and various pectin quantities.

Figure 2 presents the dynamic surface tension data of *Quillaja* saponin and *Apple* pectin mixture solutions. The saponin concentration was fixed at C(Sp) = 0.1 g/L, while pectin concentrations were varied in the range of C(Pc) = 0.0–1.0 g/L. The obtained results demonstrate the following features: (i) In the mixed solutions, and for all pectin concentrations, the surface tension values dropped smoothly up to equilibrium values in about ~5 h, while in the case of saponin-only samples (black symbols), the significant decrease was established within a shorter period of time (~3 h). (ii) In four of the *Apple* pectin concentrations in the mixtures (0.1 g/L, 0.3 g/L, 0.4 g/L and 1.0 g/L), stable equilibrium values were achieved, with the lowest values accomplished at the highest pectin quantity (C(Pc) = 1.0 g/L). (iii) In the mixed samples with C(Pc) = 0.5 g/L and 0.7 g/L, the surface tension data dropped regularly within ~ 11 h and then slightly increased, approaching the values registered in the saponin/pectin mixtures with C(Pc) = 0.4 g/L.

The experimental results give ample evidence that the addition of *Apple* pectins to saponin aqueous solutions has a significant effect on the adsorption layer properties of the mixed samples at the air/solution interface. Furthermore, it seems that the ratios 1:3 and 1:4 (g/L, by weight) are particularly suitable for the synergistic enhancement of the saponin–pectin interactions. This might be attributed to the additional formation of mixed saponin/pectin structures, most probably in the subinterface at the air/solution boundary, thus leading to restricted blockage of the adsorbed saponin entities, particularly at suitable and well-defined *Apple* pectin quantities. The possible hypothesis is that pectin might capture/fix saponin molecules, forming mixed complexes that somewhat adsorb through the saponin portions at the air/solution interface while remaining partially located in the solution subinterface due to extended pectin molecule configurations. Thus, the synergy of both bio-ingredients is experimentally well evidenced and was more pronounced at specific pectin concentrations. Upon raising the pectin quantity in the mixtures, lower surface tension values were acquired.

**Figure 3:**
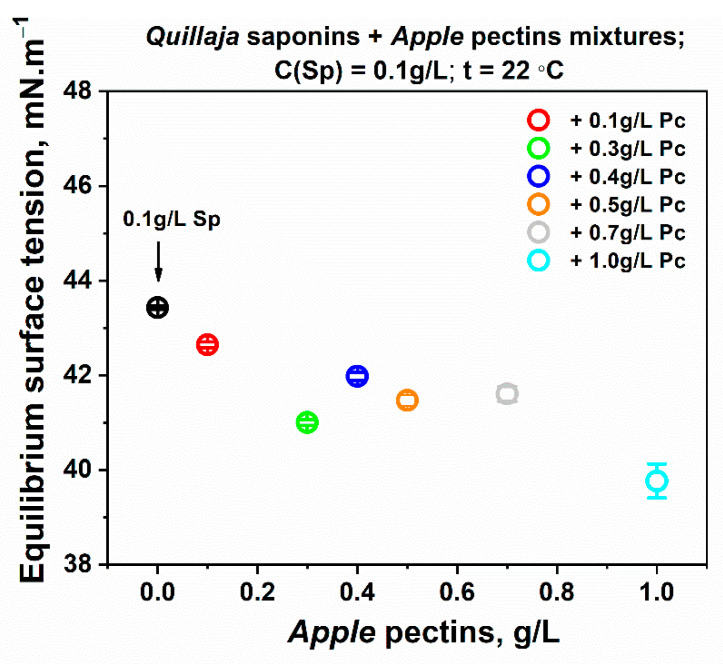
Equilibrium surface tension of *Quillaja* saponin and *Apple* pectin mixture solutions at constant saponin concentration (C(Sp) = 0.1 g/L) and at various pectin quantities.

The equilibrium surface tension values obtained in the PAT setup are presented in Figure 3. As shown, at various pectin concentrations and with constant saponin quantities, all mixed formulations demonstrated higher surface activity than in the saponin-only samples. Upon the increase of pectin quantity, this tendency was preserved with some peculiarities: there was a local minimum in the surface tension values at C(Pc) = 0.3 g/L and a peak at C(Pc) = 0.4 g/L, with an almost plateau region in-between C(Pc) = 0.3–0.7 g/L. The data might also be regarded as experimental evidence about the synergistic interactions between the two bioingredients.

#### 3.1.2. Surface Dilational Rheology

**Figure 4:**
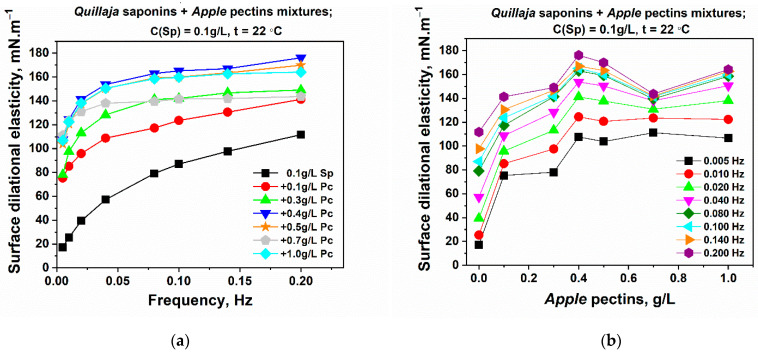
Surface dilational elasticity of adsorption layers of *Quillaja* saponin-only, and *Quillaja* saponin and *Apple* pectin mixtures at constant saponin concentration (C (Sp) = 0.1 g/L) (**a**) vs. frequency of the bubble’s deformations (**b**) vs. *Apple* pectin concentration.

The results about surface dilatational elasticities of the adsorption layers at the air/solution interface shed more light on the properties of the investigated systems (Figure 4a,b). Both frequencies and pectin concentration dependencies at a fixed saponin quantity (C(Sp) = 0.1 g/L) were investigated. The obtained values for the saponin-only sample were high (Figure 4a) and depended strongly on the bubble oscillation frequencies. This is in line with the provisional structure of the adsorption layer, with the adsorbed saponin molecules most probably attaining a “lay-on” configuration of the hydrophobic section at the air/solution interface with glycoside chains participating in hydrogen bonding interactions in the aqueous sublayer [36]. Upon the addition of more *Apple* pectins, the surface dilation elasticity values were further increased and maximum values were achieved for concentrations C(Pc) = 0.4 g/L, 0.5 g/L and 1.0 g/L. The surface dilational elasticities were slightly lower at C(Pc) = 0.3 g/L and 0.7 g/L, but still remained very high compared to the saponin-only case (Figure 4a,b).

**Figure 5:**
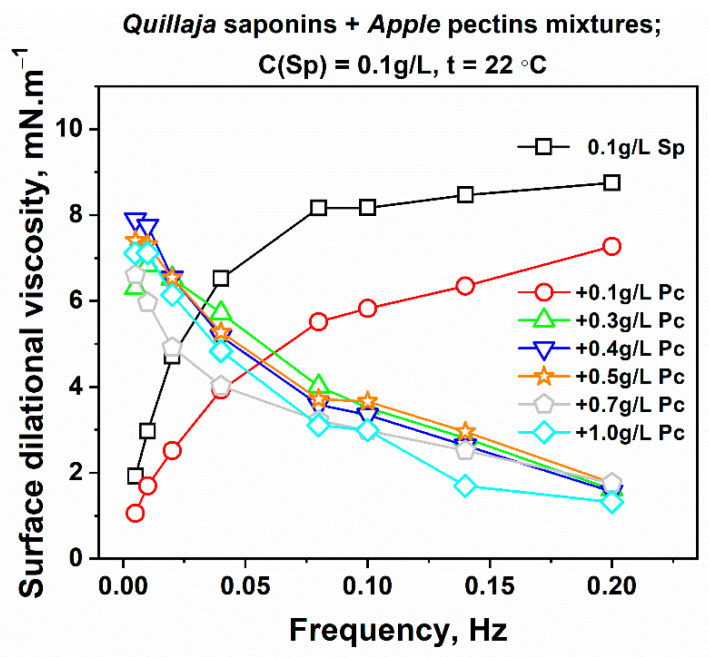
Surface dilational viscosity of adsorption layers of *Quillaja* saponin-only, and *Quillaja* saponin and *Apple* pectin mixtures at constant saponin concentration (C(Sp) = 0.1 g/L) and various *Apple* pectin concentrations against the frequency of the bubble’s deformations.

As for the surface dilational viscosities, the values were higher for the *Quillaja* saponins-only systems (Figure 5). In the cases of 1:1 mixtures (g/L, by weight), there was a detectable effect of the possible pectin/saponin synergistic interactions and the interfacial viscosities were slightly diminished compared to the saponin-only case. Upon further increase of the pectin quantity at C(Pc) ≥ 0.3 g/L, however, the adsorption layer behaved as purely elastic and the surface dilational viscosities were profoundly reduced.

### 3.2. Microscopic Foam Films

Microscopic foam films are intrinsic structural components of foam systems. Therefore, their drainage performance and stability are of considerable importance for the properties of biodegradable and biocompatible foams. Thin liquid film investigations were carried out using the microinterferometric method of Scheludko–Exerowa and the thin liquid film–pressure balanced technique. The films were obtained following a 30-min preliminary incubation period at the preferred temperature in a thermostatic chamber of the instrumentation setup (the temperature was kept at t = 22 °C).

**Figure 6:**
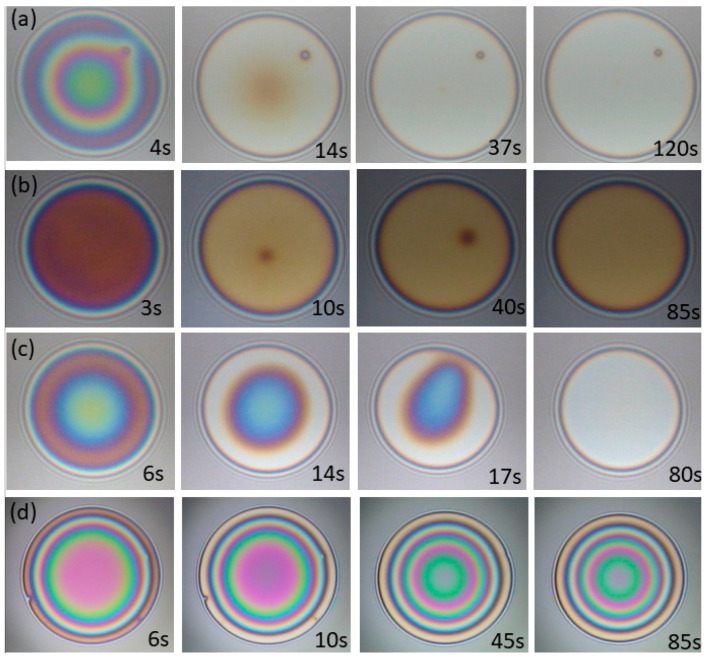
Snapshots of foam films stabilized by: (**a**) 0.1 g/L *Quillaja* saponin-only; (**b**) 0.1 g/L *Apple* pectin-only; (**c**) mixture of 0.1 g/L *Quillaja* saponins and 0.1 g/L *Apple* pectins; (**d**) mixture of 0.1 g/L *Quillaja* saponins and 0.2 g/L *Apple* pectins.

Characteristic snapshots of the foam films, taken at consecutive stages of the drainage process using a Scheludko–Exerowa cell, are presented in Figure 6. In all cases, the microscopic foam films were non-rupturing and drained slowly to equilibrium thickness values. The films stabilized by saponin-only solutions are relatively thick (Figure 6a, Table 1, h(Sp) = 98.7 ± 4.6 nm). The films from pectin-only aqueous samples were even thicker than in the saponin-only case (Figure 6b and Table 1, h(Pc) = 102.4 nm). However, at C(Sp) = 0.1 g/L and C(Pc) = 0.1 g/L, the film thickness values in 1:1 mixtures were considerably lower than in the single component systems (h= 83.4 ± 1.9 nm, Table 1). The thickness increased significantly upon the rise of the pectin quantity in 1:2 mixtures (h > 102.4 nm, Table 1). At the same time, the overall drainage performance was different: the snapshots revealed that the drainage was significantly retarded, and the films were completely nonhomogeneous but remained symmetric.

**Figure 7:**
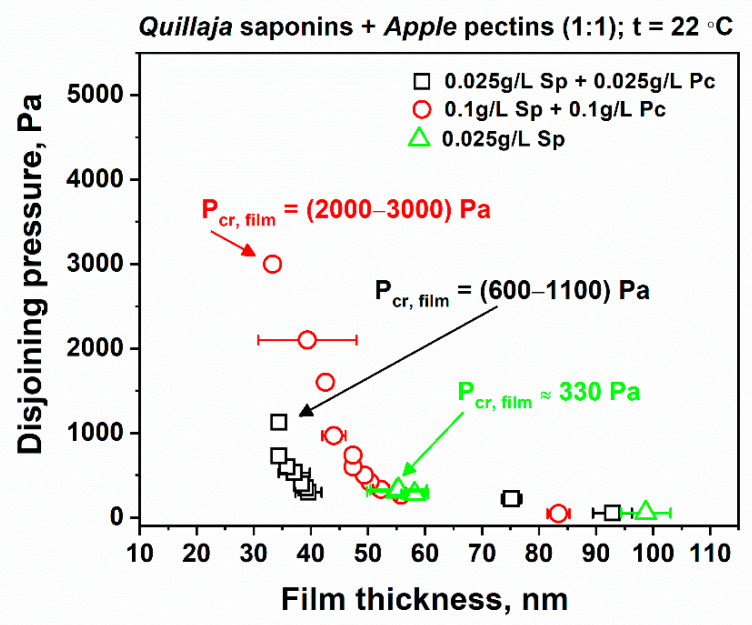
Disjoining pressure vs. film thickness isotherms of microscopic foam films from *Quillaja* saponin solution and its mixture with *Apple* pectins at 1:1 (g/L, by weight).

The disjoining pressure vs. film thickness isotherms (Π vs. h) for the microscopic films of saponin-only solutions and mixtures of *Quillaja* saponins and *Apple* pectins in the case of 1:1 (g/L, by weight) are presented in Figure 7. The measurements were performed using a porous plate and TLF-PBT instrumentation. Two concentrations of the saponin component were investigated: 0.025 g/L and 0.1 g/L. At the lower saponin-only concentration (C(Sp) = 0.025 g/L), the film thickness decreased upon the rise of the pressure. The films gradually drained in the range of h = 92.87 ± 3.4 nm to h = 55.3 ± 5.0 nm, and the critical pressure of film rupture was P_cr,film_ ≈ 330 Pa. On the contrary, at the higher concentration of the saponin-only case (0.1 g/L), the foam films were more unstable and ruptured at the pressure of their formation; thus, the run of Π vs. h could not be obtained.

The addition of *Apple* pectins to the aqueous solution tends to stabilize the microscopic foam films. As is shown in Figure 7, the films from mixed solutions of saponin and pectin drain up to ~33–35 nm and rupture at high pressure. Furthermore, the higher total concentration of the mixed systems favored the film stabilization and P_cr,film_ increased from 600–1100 Pa (0.025 g/L Sp + 0.025 g/L Pc) to 2000–3000 Pa (0.1 g/L Sp + 0.1 g/L Pc). Considering the previously established correlation between the foam film critical pressure of rupture and the stability of the corresponding foams [23,35], it is to be expected that foam systems obtained from mixed saponin and pectin formulations should be more stable than those from single-component saponin-only solutions.

**Figure 8:**
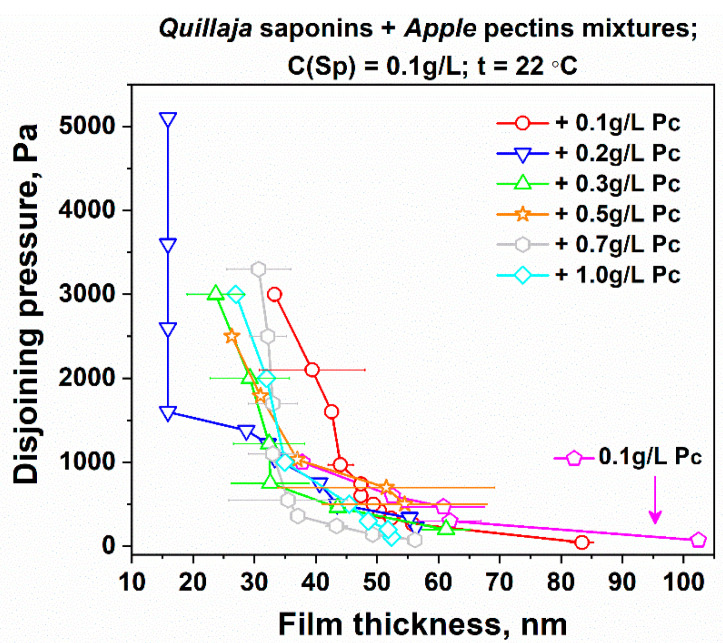
Disjoining pressure vs. film thickness isotherms of foam films from *Quillaja* saponin solution at C(Sp) = 0.1 g/L and its mixtures with various *Apple* pectin quantities. The isotherm for the *Apple* pectin-only solution at C(Pc) = 0.1 g/L is also presented.

In order to examine the validity of this idea, the disjoining pressure isotherms for the mixed solutions at a constant saponin concentration of 0.1 g/L and at various pectin quantities are presented in more detail (Figure 8). Note that the respective data about the disjoining pressure isotherm for microscopic foam film from pectin-only samples at C(Pc) = 0.1 g/L run in a similar manner as in the case of saponin-only systems and P_cr,film_ ≈ 220 Pa. The obtained results for the critical pressure of film rupture are summarized in Table 2.

It is notable that despite the different system compositions, the curves and the thinning rates run in a similar manner. Generally, upon the increase of pectin concentration, the film stability is substantially increased as compared to the single component samples (Figure 8). Higher pectin concentrations tend to favor foam film stabilization at lower thickness values. However, it could be seen that the maximum stabilization was achieved at a particular pectin quantity, namely C(Pc) = 0.2 g/L, and results in a minimum film thickness: h = 16 nm (see also Table 2). Therefore, it might be presumed that in this specific sample composition, the synergistic interactions of both components are optimal in view of the foam film stabilization procedure.

## 4. Discussion

The major outcomes from the study may be summarized as follows:

1. Adsorption layer properties of aqueous solutions containing mixed formulations of *Quillaja* saponins and *Apple* pectins give plenty of indications for the onset of synergistic interactions in the investigated systems. The synergy is enhanced upon the increase of the pectin quantity at a fixed quantity of saponin (e.g., C(Sp) = 0.1 g/L, Figure 2). However, maximum effects are achieved at specific pectin concentrations. For example, surface dilational elasticity at the air/solution interface of the mixture is almost doubled at C(Pc) = 0.4, 0.5 and 1.0 g/L, as compared to the intrinsically high saponin-only solutions (Figure 4a,b), while the surface dilational viscosity is substantially decreased for all pectin quantities and there is no substantial dependence on the frequencies of the bubble’s deformation (Figure 5). Another interesting peculiarity is that the elasticity values at the highest frequencies are grouped in two tendencies: the maximum values are related to pectin additions of C(Pc) = 0.4, 0.5 and 1.0 g/L, while the next step (lower values) is registered at C(Pc) = 0.1, 0.3, 0.7 g/L of *Apple* pectin additions to the saponin aqueous solutions.
2. The results on the drainage behavior and the stability of microscopic foam films support the notion of complex synergistic interactions in the mixed solutions. Thus, at a given saponin concentration, the foam films are significantly stabilized by the addition of various *Apple* pectin quantities. There exist optimum ratios of the components in the aqueous solution formulations, at which maximum synergy effects are observed: at C(Sp) = 0.1 g/L and C(Pc) = 0.2 g/L, the highest stability of the foam films is registered (Figure 8).

Therefore, the combined surface tension measurements and thin liquid film studies provide important hints about complex interaction interchange with possible structural reorganization at the air/solution interface and in the subinterfacial area of the aqueous systems. In order to understand these outcomes, it is necessary to account for the specific properties of the single-component aqueous solutions.

**Figure 9:**
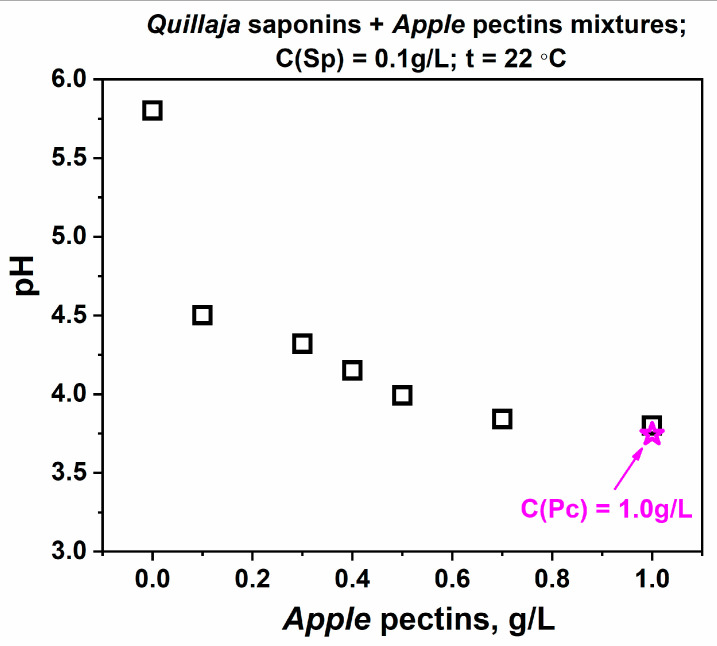
pH of *Quillaja* saponin and *Apple* pectin mixture solutions at constant saponin concentration (C(Sp) = 0.1 g/L) vs. various pectin concentrations. The pH value of the *Apple* pectin-only solution at C(Pc) = 1.0 g/L is also presented.

First, it is known that *Quillaja* saponins have high surface activity and exhibit considerable foaming properties [2,37]. The conformation of the bidesmosidic *Quillaja* saponins usually induces a “lay-on” configuration arranged in the adsorption layer at the air/solution interface [36,37]. Thus, the sugar chains are facing the water phase and are involved in a network of H-bonding interactions while leaving the aglycone orientated parallel to the interface. This peculiarity is the primary reason for the exceptionally high surface dilational values of the adsorption layer in the saponin-only samples, as well. Although saponins are expected to behave as slightly anionic surfactants in aqueous solutions, it was established that the particular type of *Quillaja* saponin molecules investigated here, at ambient conditions and without specific pH regulation, might be considered as nonionic surfactants within the range of pH = 4.3–6.4 [23], which is the case in the study here (Figure 9).

Second, while *Apple* pectins have both hydrophilic and hydrophobic portions, within the range of concentrations studied here, pectin-only samples exhibit no surface activity at the air/solution interface (see Figure 1). It is well-known that pectin molecules tend to form gels in aqueous media, enhancing the bulk viscosity in the systems [12,13,14,18,19,20,38,39]. The gel formation is initiated by the intramolecular reorganization in the solution bulk in such a way that the hydrophilic portions are directed toward the water surroundings. For example, it was demonstrated that high methoxide pectins (HMP) are strong gellants at low pH (pH < 3.5) [22].The stability of the gel structure is governed predominantly by hydrogen-bonding and hydrophobic interactions, the latter being affected by the presence of sugars or similar co-solutes [40].

In order to monitor the pH values of the investigated mixed formulations and their possible impact on the synergistic phenomena, we have measured the pH of the samples at fixed *Quillaja* saponin concentrations (C(Sp) = 0.1 g/L), and upon addition of various quantities of the *Apple* pectins. The results are presented in Figure 9. As is to be seen, the pH of the mixtures was sharply decreased at a ratio of 1:1 (g/L, by weight) as compared to saponin-only solutions (from pH = 5.7 up to pH = 4.5) and changed only slightly up to the components’ ratio 1:7 (g/L, by weight). Then it remained constant at pH ~ 3.7 up to C(Sp): C(Pc) = 1:10 (g/L, by weight). Thus, the synergistic effects in the solutions containing both ingredients should be more pronounced at higher pectin concentrations (e.g., C (Pc) > 0.4 g/L), where lower pH values of the mixed formulations are systematically registered. 

To summarize the outcomes of the present study: upon addition of *Apple* pectins, several distinct peculiarities are registered. While for saponin-only solutions, the surface tension is slightly increased following the achievement of ‘equilibrium’ values, such effect is not observed at C(Sp) = 0.1 g/L and C(Pc) = 0.1 g/L, and the equilibrium data remain stable and lower than in the saponin-only sample (Figure 2). One plausible hypothesis is the additional formation of a subinterfacial layer of stretched pectin molecules. The extended configuration may be due to the participation of pectin molecules in the network of H-bonding interactions initiated by the sugar chains of the adsorbed saponins and thus complementing the structural stability of the adsorption layer. The possible outcome of such a stabilization mechanism can be followed upon further increase of the pectin quantity. Thus, at fixed C(Sp) = 0.1 g/L and C(Pc)> 0.1 g/L, the dynamic surface tension curves tend to attain a stable equilibrium surface tension value, lower than in the saponin-only samples. Another descent of the surface tension values is observed at C(Pc) = 1.0 g/L. This step-wise decrease of the equilibrium surface tensions may be related to the sequential reorganization of the subinterfacial region upon the increase of C(Pc) due to the enhancement of the gel-formation tendency in the solution bulk and the resulting restructuring of the adsorption layer coverage. Surface dilational elasticity data run in synchrony with these notions: at C(Sp) = 0.1 g/L, maximum values are achieved at specific pectin quantities of C(Pc) = 0.4, 0.5, 1.0 g/L, while lower values are registered at C(Pc) = 0.3, 0.7 g/L in the mixtures.

The surface dilational viscosity of the saponin-only samples is measurable and reaches plateaus at higher frequencies (≥ 0.10 Hz). Adding *Apple* pectins at C(Pc) = 0.1 g/L only slightly decreased it. However, the surface dilational viscosities drop to insignificant values at the increase of the pectin quantities, particularly at higher frequencies (≥0.10 Hz). This is additional evidence that the major synergistic interactions are related predominantly to the air/solution subinterface reorganization and not to the initial saponin-determined structure of the adsorption layer. Within the investigated concentration ratios, the pectin molecules are involved in the reorganization in the subsurface region, contributing to the H-bonding network, while the interfacial coverage is dominated by the saponins’ patches, which are fixed in number but are engaged in interaction with the pectin molecules of the subinterfacial region. Due to the considerable difference in the mean molecular weight of the components, one might assume the hypothesis that there is a considerable synergy in the mixed solutions. Both, *Quillaja* saponins—through stretching in the subinterfacial region, and *Apple* pectins—through shrinkage of the adsorption area of the patches in the adsorption layer—contribute actively to the adsorption layer reorganization at the air/solution interface in the mixed systems.

**Scheme 1:**
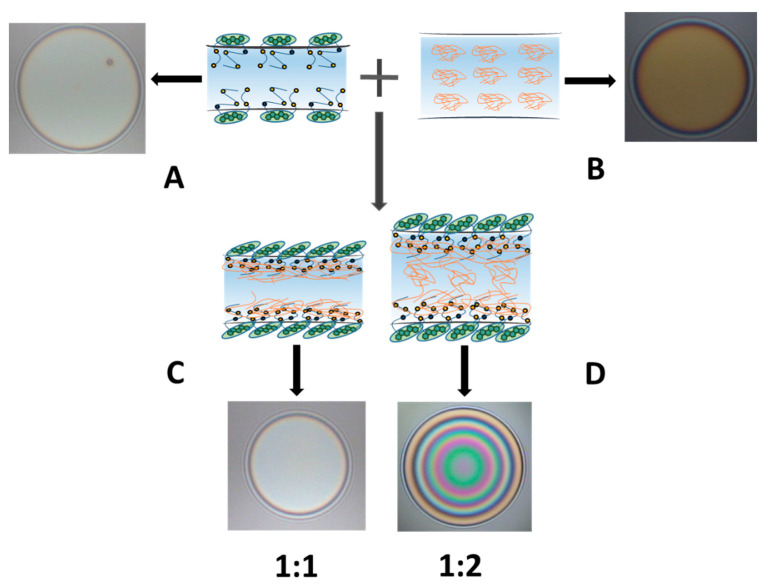
Schematic sketches illustrating the hypotheses about the structure of the subinterfacial regions and the microscopic foam films from: (**A**) saponin-only; (**B**) pectin-only; (**C**) 1:1 mixed saponin:pectin solution samples; (**D**) 1:2 mixed saponin:pectin solution samples.

The qualitative foam film results (Figure 6) provide supplementary verifications for the advanced hypotheses. It is established that all single-component and mixture solutions form stable films during the measurements in the Scheludko–Exerowa cell. However, the mechanism of the film stabilization in various cases is different. In the saponin-only samples, the basic cause for the onset of stable films is the adsorbed saponin molecules, and the hydrogen-bonding intermolecular interactions of the sugar residues dipped into the water environment (Scheme 1A). The pectin-only solutions at the studied concentration range do not exhibit measurable surface activity at the air/solution interface. However, thick stable foam films are formed (Scheme 1B). One possible reason is that because of the gel-structuring in the constrained volume of aqueous film, the bulk is additionally activated.

In the case of *Quillaja* saponin and *Apple* pectin aqueous mixtures, there is a considerable synergy, and it may be considered that both ingredients contribute to the stabilization of the microscopic foam films. The presence of high molecular mass *Apple* pectins, with well-expressed bulk gelation performance [38,39,40], interfere with the impact of the surface active saponin molecules, resulting in characteristic and very symmetric qualitative pictures of the microscopic foam films in the Scheludko–Exerowa cell (Scheme 1).

The studies of disjoining pressure vs. film thickness isotherms reveal more details that might be related to the advanced hypotheses (Figure 7 and Figure 8). In the cases of single-component systems, the critical pressure of film rupture was very low: P_cr,film_ ~330 Pa for saponin-only samples (C(Sp) = 0.1 g/L) and P_cr,film_ ~220 Pa for pectin-only solutions (C(Pc) = 0.1 g/L) (Table 2). Furthermore, in the mixed systems and at a saponin concentration of C(Sp) = 0.1 g/L, the critical pressure of rupture was generally not sensitive to the quantity of pectin and P_cr,film_ ~ 3000 Pa; however, the film thickness of rupture was substantially reduced upon raising pectin quantity (Figure 8). This decrease, however, was not regular but the respective critical thickness values alternated upon the increase of the added pectin. This performance of the film properties is reminiscent of changes in the surface dilational rheological properties and presents additional evidence about the reorganizations of the subinterfacial region and possibly of the adsorption layer coverage upon the changes in the pectin concentration (see also Scheme 1C,D).

Two key outcomes related to the stability of the mixed systems are to be accentuated: (i) Addition of *Apple* pectins to saponin aqueous solutions always enhances the film stability and results in the formation of well-structured foam films, which can rupture only at high disjoining pressures. (ii) The very stable but thinnest films are obtained at a specific content of the mixed solution, namely C(Sp) = 0.1 g/L and C(Pc) = 0.2 g/L, with P_cr,film_~5000 Pa (Figure 8). This saponin:pectin ratio (1:2 in g/L, by weight) is obviously optimal in view of the maximum stabilization of the foam films.

## 5. Conclusions

To conclude, the obtained results show that there are optimum weight ratios of saponins:pectin quantities which result in enhanced synergistic interactions and restructuring of the aqueous solution formulations in view of stabilization of the adsorption layers and of the thin liquid films in foam systems. Generally, the obtained experimental results back up the hypothesis that at a given *Quillaja* saponin content, there is a narrow range of *Apple* pectin quantity where a well-expressed synergy between the two bioactive ingredients of natural origin in aqueous solution formulations is registered. This synergy initiates intermolecular interactions leading to particular bulk sublayer reorganization and excessive adsorption layer stability, related primarily to high surface dilational elasticity values in foam systems. When two such interfaces come nearer and form a microscopic liquid film, the specific complex bulk structures in the film bulk also interact, resulting in very stable foam films. The latter effect is observed at well-defined content of the two bioactive ingredients (1:2), which is different from the values of maximum enhancement of the single adsorption layer stability values (1:1).

More studies are planned, particularly on foam systems obtained from aqueous solution formulations involving these bioactive ingredients. For example, one important issue is the fine-tuning foam formation and stability by the addition of low-molecular mass (LMM) electrolytes and/or through mild temperature changes that are expected to have a considerable impact on the hydrogen-bonding network and the bulk gelation performance.

Therefore, the presented study and the obtained data constitute an essential basis for further paths of optimizing the aqueous compositions and fine-tuning the properties of bio-compatible foams based on the bioactive ingredients *Quillaja* saponin and *Apple* pectin in view of future industrial, pharmaceutical and biomedical applications.

## Figures and Tables

**Table 1:** Film thickness of foam films from single component and mixture solutions, obtained by the use of a Scheludko–Exerowa cell.

*Quillaja* Saponins, C(Sp), g/L	*Apple* Pectins, C(Pc), g/L	Film Thickness h, nm
0.025	-	98.7 ± 4.4
0.025	0.025	92.8 ± 3.4
0.1	-	98.7 ± 4.6
-	0.1	102.4
0.1	0.1	83.4 ± 1.9
0.1	0.2	>102.4

**Table 2:** Critical pressure of rupture of foam films from aqueous solutions of saponin-only, pectin-only and saponin/pectin mixtures, obtained using porous plate in TLF-PBT.

*Quillaja* Saponins C(Sp), g/L	*Apple* Pectins C(Pc), g/L	Critical Pressure of Film Rupture P_cr,film_, Pa
0.025	-	330
0.025	0.025	600–1000
0.1	-	220
-	0.1	400–1000
0.1	0.1	2000–3000
0.1	0.2	1000–5000
0.1	0.3	2000–3600
0.1	0.5	500–2500
0.1	0.7	2700–3700
0.1	1.0	500–3000

## Data Availability

Data are available upon request.
